# Case report: A case of *SLC26A4* mutations causing pendred syndrome and non-cystic fibrosis bronchiectasis

**DOI:** 10.3389/fped.2022.1077878

**Published:** 2023-01-09

**Authors:** Kang Zhu, Yingkang Jin

**Affiliations:** Department of Respiratory, Guangzhou Women and Children's Medical Center, Guangzhou Medical University, Guangzhou, China

**Keywords:** Bronchiectasis, pendred syndrome, gene mutations, cystic fibrosis, SLC26A4

## Abstract

The *SLC26A4* gene encodes the transmembrane protein pendrin, which is involved in the ion transport of chloride (Cl^-^), iodide (I^-^) or bicarbonate (HCO3^-^). Mutations in the *SLC26A4* gene alter the structure and (or) function of pendrin, which are closely related to Pendred syndrome. What’s more, researchers have demonstrated in vitro that mutations of *SLC26A4* cause acidification of airway surface fluid (ASL), reduce airway defense, and increase the thickness of ASL. In the context of infection, it may lead to chronic inflammation, destruction of airway wall architecture and bronchiectasis. However, there is no case report of bronchiectasis caused by *SLC26A4* gene mutations. Here, we describe the first case of Pendred syndrome and non-cystic fibrosis bronchiectasis in a child possibly caused by *SLC26A4* mutations. We remind clinicians to pay attention to the possibility of bronchiectasis in patients with *SLC26A4* gene mutations.

## Introduction

Bronchiectasis is a chronic pulmonary disorder characterized by chronic cough, sputum formation and recurrent pulmonary exacerbations. The causes of bronchiectasis are very various. It could be simply divided into bronchiectasis caused by cystic fibrosis (CF) and bronchiectasis caused by non-CF. The common causes of non-CF bronchiectasis include infections (e.g., tuberculosis, whooping cough), gastroesophageal reflux disease (GERD), foreign bodies, alpha1-antitrypsin deficiency, autoimmune diseases, immunodeficiency disorders, primary ciliary dyskinesia (PCD), and excessive exposure to toxic gases (1). The factors mentioned above may cause chronic airway inflammation and impaired mucociliary clearance, and ultimately lead to mucus accumulation and airway structural disruption (2). Once this process is established, it will become a vicious circle over time.

The *SLC26A4* gene encodes pendrin which is expressed in the inner ear, thyroid, kidney and airways. Pendrin is responsible for transporting many ions across cell membranes, including chloride (Cl^−^), bicarbonate (HCO3^−^), hydroxyl ion (OH^−^), iodide (I^−^) or thiocyanate (SCN^−^) (3). Dysfunction of pendrin is closely related to Pendred syndrome, which is characterized by sensorineural deafness, inner ear malformation and goiter (4). In airway epithelium, pendrin is expressed mainly in secretory non-ciliated cells and mediates the transport of Cl^−^ against HCO3^−^ or SCN^−^ (5). What's more, some researchers have observed a functional coupling between pendrin and cystic fibrosis transmembrane conductance regulator (CFTR), a very important transporter of Cl^−^ in airway epithelium (6, 7). Studies *in vitro* showed that the loss of pendrin function could affect Cl^−^/HCO3^−^ exchange, resulting in a decrease in pH value and an increase in the thickness of airway surface fluid (ASL) (8, 9). Bicarbonate in ASL facilitates mucin unpacking and bacterial killing, in other words, the low pH of ASL decreases the ability to kill the trapped bacteria and initiates host defense abnormalities (10). Chronic inflammation and the increase of ASL thickness caused by pendrin dysfunction may lead to pathological features of bronchiectasis. However, as far as we know, there is no case report of bronchiectasis caused by *SLC26A4* gene mutations. Here, we report the first case of Pendred syndrome and non-CF bronchiectasis in a child possibly caused by mutations in the *SLC26A4* gene, and alert physicians to the possibility of bronchiectasis caused by *SLC26A4* mutations. This report may enrich the etiology of bronchiectasis.

## Case presentation

**Figure 1:**
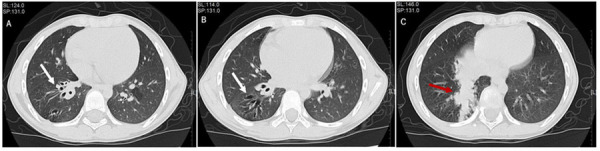
Features of a high-resolution computed tomography scan of the chest of this child. (**A,B**) It showed dilatation of the trachea in the right lower lobe bronchus. (**C**) It revealed airway mucus obstruction.

A 7-year-old boy was taken to the hospital by his parents due to an accidental impact on his abdomen while playing together with his classmates. His physician arranged an abdominal computed tomography (CT) examination for him. In the process of abdominal CT scanning, the examiner found no obvious abnormal signs in his abdomen. However, he accidentally found that the child's lower lobes of right lung had some signs of airway dilatation and mucus retention, and thus the examiner reported these findings responsibly to the doctor in charge. Next, the child was transferred to the respiratory specialist in our hospital and completed a chest high-resolution computed tomography (HRCT) scan. The HRCT revealed multiple dilations and mucus plugs in the right lower lobe bronchus (Figures 1A–C). The dilated trachea was approximately 7 mm in diameter at its widest point. Multiple patchy shadows with ill-defined borders were seen in the right lower lobe, mainly in the medial and posterior basal segments. In addition to that, several well-demarcated, irregular, partial translucent shadows were seen in the left lung. The right pleura was slightly thicker. According to the published guidelines, the child was radiologically diagnosed with bronchiectasis (11). However, we had admitted the patient without any respiratory symptoms. During auscultation, we only found that the breath sound of the right lower lung was mild decreased.

In order to find out the cause of bronchiectasis and treat it, we asked in detail about clinical symptoms and past medical history, and conducted a series of tests. Initially, we asked about the child's previous pathological symptoms. The caregiver clearly stated that the child did not have recurrent cough, expectoration, hemoptysis, chest pain, bloody sputum, fever, night sweats, fatigue, weight loss, nausea, acid reflux, heartburn, postnasal drip, salty skin or sinusitis. His growth and development were similar to those of his peers, but his athletic endurance was not as good as theirs. He had no history of tuberculosis, measles, whooping cough, GERD and hospitalization for respiratory infection, nor had he been exposed to pollution and toxic gases. He had only one history of mild and chronic cough, and the symptoms lasted for about 1 month when he was four years old. After several outpatient visits and taking oral medication, he gradually recovered. A pediatric chest x-ray showed inflammation in the right lower lung, but no signs of bronchiectasis. He did not undergo a HRCT of the chest at that time. What's more, he had a history of Pendred syndrome. He suffered from hearing loss at the age of 2 and received a cochlear implant surgery at the age of 3 and a half. He had a mild enlarged goiter, but no thyroid dysfunction.

**Figure 2:**
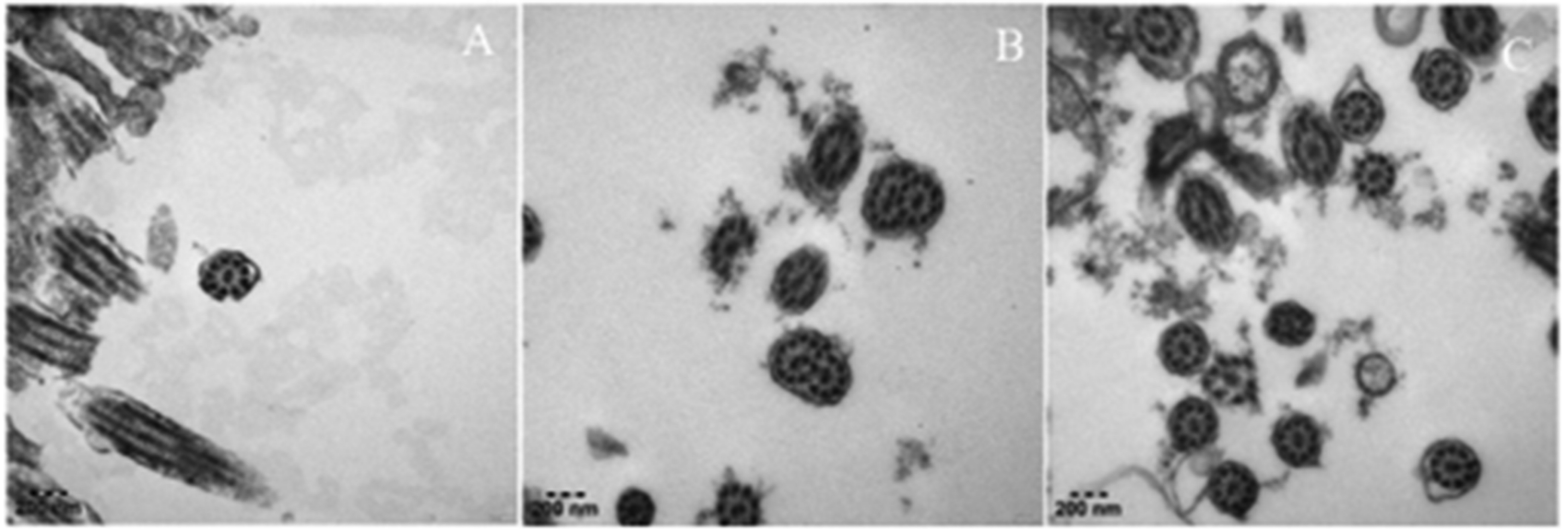
Ultrastructure of respiratory cilia in this child. (**A–C**) It showed normal ciliary ultrastructure: cross-section of cilia showing 9 + 2 doublet microtubule.

We performed extensive diagnostic tests on this child. A complete blood count, C-reactive protein, results of kidney- and liver- function tests, serum procalcitonin, tuberculin test and interferon-gamma release assays were normal. A test for antineutrophil cytoplasmic antibody was negative, as were tests for some viruses which could cause secondary immunodeficiency, such as human immunodeficiency virus. Immunoglobulin level and absolute lymphoblastic counts in serum were also normal. The detection of allergen-specific IgE revealed a mild positive reaction to dust mites. Moreover, we performed fiberoptic bronchoscopy, bronchoalveolar lavage (BALF), and bronchial biopsy. Bronchoscopy revealed condensed secretions in the right lower lobe bronchus. The results of next-generation sequencing of BALF revealed Tropheryma whipplei (sequence number 13,200). Electron microscopy of bronchial biopsy showed mild inflammatory changes in cilia, without typical ultrastructural pathological changes of PCD (Figures 2A–C). We also tested for CF and *α*1-antitrypsin deficiency related mutant genes. These results were negative. Because infection was the most common cause of bronchiectasis, and a large number of bacteria called Tropheryma whipplei were shown in BALF, we first considered whether bronchiectasis was caused by Whipple's disease. Common signs and symptoms of Whipple's disease included diarrhea, stomach cramping and pain, inflamed joints, weakness, and anemia. Obviously, the child did not meet the diagnosis criteria. Secondly, we analyzed other common causes of bronchiectasis. The evidence for the diagnosis of the above-mentioned causes of bronchiectasis in this child was also insufficient. So far, a definitive diagnosis of the child has not been determined.

**Figure 3:**
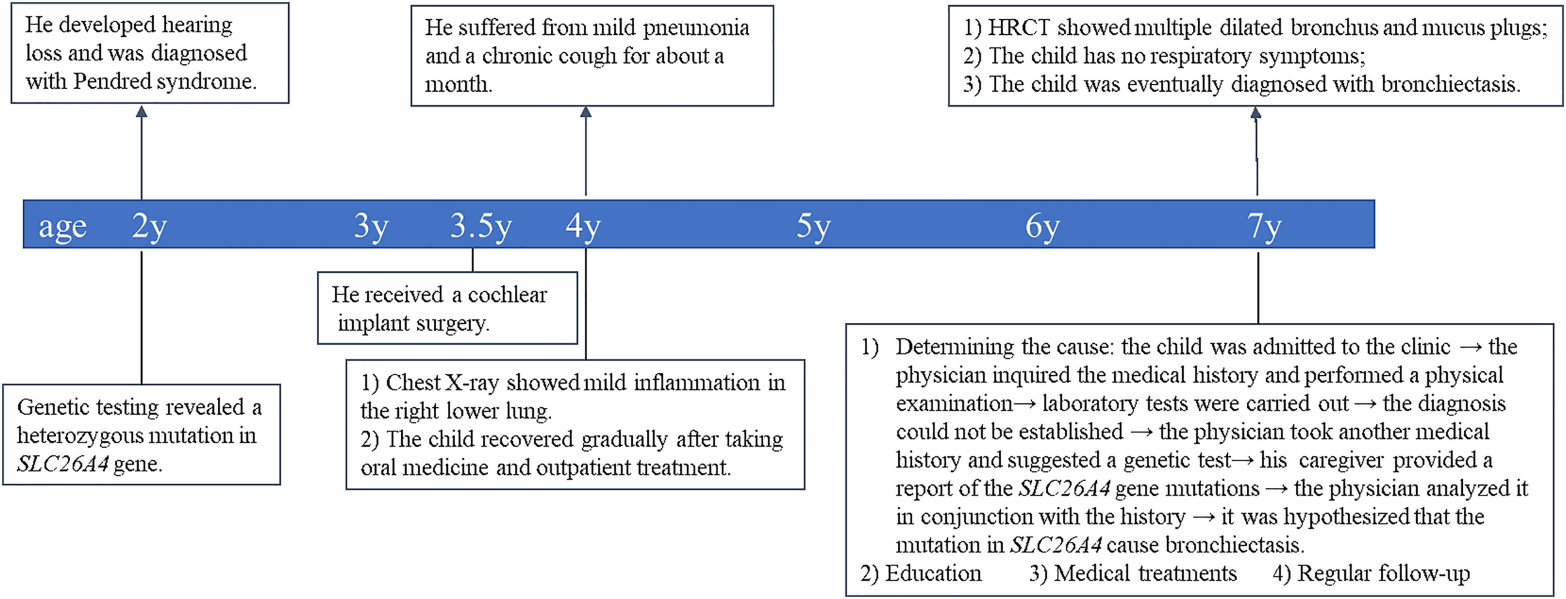
Timeline of clinical events, diagnostics, and treatments of the patient.

Considering the importance of medical history in making an accurate diagnosis, we inquired the child's medical history again and recommended a genetic test for this child. The caregiver supplemented the medical history and stated that the child was found to have hearing loss at the age of 2. Genetic testing for hearing loss showed a heterozygous mutation in the *SLC26A4* gene, and the mutation site was IVS7–2A > G (Table 1). We learned that IVS7–2A > G may lead to the deletion of exons surrounding the splice site. Mutations in the *SLC26A4* gene alter the structure or function of pendrin, which disrupts ion transport. In the inner ear, pendrin dysfunction affects Cl^−^ transport, leading to vestibular aqueduct enlargement and sensorineural hearing loss (12). Goiter is mainly related to the reduction of pendrin-mediated transport of I^−^ (13). Pendrin also plays an important role in airway defense and airway homeostasis *in vitro*. We questioned the link between *SLC26A4* mutation and bronchiectasis. The loss of pendrin function could cause airway acidification, impair airway epithelial defense, and increase of the volume of ASL. In the case of infection (even opportunistic pathogens), patients may not be able to effectively eliminate the infection, but show persistent chronic infection and inflammation. Chronic airway inflammation and airway blockage interact with each other, and finally lead to irreversible tracheal dilatation. Based on the above analysis, we speculated that mutations in the *SLC26A4* (pendrin) may cause non-CF bronchiectasis. Unfortunately, our conclusions were speculative. And the proposed mechanism has not been verified by experimental researches. To the best of our knowledge, no similar cases have been reported in other clinical studies. The root causes of this phenomenon is worth further exploration. We explained the analysis and speculative conclusions to the patient's family members and get their recognition. Bronchiectasis possibly caused by pendrin dysfunction, like bronchiectasis caused by other causes, may be currently incurable. However, studies have shown that the expression of pendrin in the endolymphatic sac can be successfully restored in mice by transgenic technology (14). Perhaps the function of pendrin in the airway could be restored in the future. Therefore, we gave the child a series of treatments, including inhalation of bronchodilators and corticosteroids, taking oral apophlegmatisant and antibiotics, and mechanical vibration expectoration. And he was taught to avoid respiratory tract infection, get the pneumonia vaccine, master the ability of airway clearance and regularly follow up. After 1 month of treatment, the child returned to our outpatient clinic and performed a CT of the chest. The images showed that the inflammation disappeared, but the tracheal dilatation remained. Figure 3 showed timeline of clinical events, diagnostics, and treatments of the patient.

**Table 1:** The result of the mutations of the hearing loss related gene in this child. It showed a heterozygous mutation in the *SLC26A4* gene, and the mutation site was IVS7-2A > G. WT, wild type; het, heterozygous.

Mutated gene	Nucleotide change	Mutation type
GJB2	35del G	WT
		176-191del 16	WT
		235del C	WT
		299-300del AT	WT
GJB3	538C > T	WT
		547G > A	WT
SLC26A4	IVS7-2A > G	het
		2168A > G	WT
		IVS15 + 5G > A	WT
		1174A > T	WT
		1226G > A	WT
		1229C > T	WT
		1975G > C	WT
		2027T > A	WT
12S rRNA	1494C > T	WT
		1555A > G	WT

## Discussion

Bronchiectasis is a chronic disease characterized by a persistent cough and excessive secretions in the airways. Published data have reported a wide range of prevalence rates, ranging from 0.2 to 735.0 cases per 100,000 children (1). Causes of bronchiectasis are broad and diverse, including idiopathic, acquired, or infection-related lung disease. Its pathogenesis is also very complex. The defective host defense allows microorganisms to settle and colonize in the airway epithelium, leading to chronic inflammation and lung damage. The interaction between infection and inflammation may form a vicious circle, leading to impaired mucociliary clearance, structural lung damage, and accompanying clinical symptoms (15). Although the etiology and pathogenesis of bronchiectasis have been extensively studied, bronchiectasis possibly caused by mutations of *SLC26A4* gene has not been reported. Here, we report the first case of Pendred syndrome and non-CF bronchiectasis in a child possibly caused by *SLC26A4* gene mutations. We hypothesize that in the context of chronic infection and inflammation, mutations in the SLC26A4 gene may lead to acidification of the ASL, impairment of airway defenses, increased ASL volume and ultimately irreversible bronchiectasis. However, it should be noted that this is a speculative conclusion. And the rationality of this hypothesis still needs to be tested in the future.

In addition, there is an interesting topic that deserves to be explored in depth. Previous studies have confirmed the important role of pendrin in airway epithelium. IVS7–2A > G, the mutant site reported in this case, is a hotspot mutation in East Asian deaf populations (16). One question remains to be addressed Why are there no similar cases reported in other articles, and why is bronchiectasis of this child not diffuse? We speculate that, on the one hand, previous studies have not reached a consistent conclusion on the relative roles of CFTR and pendrin in HCO3^−^ secretion. One study has shown that pendrin plays a more important role in the secretion of HCO3^−^, compared with CFTR (17). Another study comes to the opposite conclusion (18). The extent of impairment of airway defenses in this patient is unclear. Perhaps, compared to pendrin, CFTR is more important for maintaining the secretion of HCO3^−^ and the defense of the airway. Thus, for patients with normal CFTR function and pendrin dysfunction, their airways may still have a certain degree of defense ability against respiratory infections, and thus do not show diffuse bronchiectasis. On the other hand, we coincidentally found that the site of infection showed in chest x-ray films at the age of 4 was consistent with the site of bronchiectasis. We guess that preexisting chronic respiratory infection may be an important inducer of bronchiectasis. Therefore, similar cases have not been widely reported. But these speculations need to be confirmed by further researches. Another intriguing phenomenon is why the child has no history of recurrent respiratory infections and physicians hear only diminished breath sounds of the lungs. This may be because the child's bronchiectasis is relatively mild and it has not yet developed a serious infection. Despite the absence of any respiratory symptoms, the child's exercise tolerance has declined, and many pathogenic microorganisms are present in his BALF.

There is no cure for bronchiectasis at present, but treatment options may help reduce the frequency of exacerbations, improve quality of life and prevent disease progression. Treatment options may include medication, vaccinations to prevent infection, physical therapy, pulmonary rehabilitation, oxygen therapy, airway clearance techniques, and surgery (19). Some studies yet found that early bronchiectasis may be reversible, especially in children and young adults who have received qualified medical service before the occurrence of destructive changes in the musculo-elastic tissues of the bronchial walls (1, 20). Early diagnosis and preventive measures are essential. We mild recommend that patients with hearing loss or Pendred syndrome due to pendrin dysfunction undergo CT scans of the chest to screen for possible early bronchiectasis, especially in people who are at high risk for respiratory infections, and those with a history of pneumonia (even if it is mild) and chronic cough. In the future, transgenic technology may cure this disease.

In conclusion, this report describes the first case of Pendred syndrome and non-CF bronchiectasis in children possibly caused by *SLC26A4* gene mutations. Clinicians are advised to be aware of the potential non-CF bronchiectasis and perform CT scans in patients with *SLC26A4* gene mutations, especially in high-risk patients. Early and periodic screening, diagnosis, and treatment are crucial for preventing the progression of lung disease.

## Data Availability

'The original contributions presented in the study are included in the article/Supplementary materials, further inquiries can be directed to the corresponding author/s.
